# Prognosis after autologous peripheral blood stem cell transplantation for osteonecrosis of the femoral head in the pre-collapse stage: a retrospective cohort study

**DOI:** 10.1186/s13287-020-01595-w

**Published:** 2020-02-26

**Authors:** Jiafei Pan, Quanwei Ding, Shuaijie Lv, Bingjiang Xia, Hongting Jin, Di Chen, Luwei Xiao, Peijian Tong

**Affiliations:** 1 Tongde Hospital of Zhejiang Province, affiliated with Zhejiang Chinese Medicine University, Hangzhou, 310012 People’s Republic of China; 2 Zhejiang Chinese Medicine University, Hangzhou, 310053 People’s Republic of China; 3 Hangzhou Fuyang Hospital of Traditional Chinese Medicine Orthopedics and Traumatology, Hangzhou, 311400 People’s Republic of China; 4 The First Affiliated Hospital of Zhejiang Chinese Medicine University, Hangzhou, 310006 People’s Republic of China; 5 Shaoxing Hospital of Traditional Chinese Medicine, Shaoxing, 312000 People’s Republic of China; 6 Institute of Orthopedics and Traumatology of Zhejiang Province, Hangzhou, 310053 People’s Republic of China; 7 Rush University Medical Center, Chicago, IL 60612 USA

**Keywords:** Femur head necrosis, Peripheral blood stem cell, Femoral head survival, Proportional hazards model

## Abstract

**Objectives:**

Autologous peripheral blood stem cell (auto-PBSC) transplantation is an effective therapeutic for the osteonecrosis of the femoral head (ONFH) but without prognosis estimation. This study mainly aimed to (1) determine whether auto-PBSC transplantation is a promising option, (2) assess the risk of hip-preservation failure, (3) achieve a predictive model of femoral head survival after the intervention, and (4) eventually identify clinical indications for auto-PBSC transplantation in future.

**Methods:**

After reviewing the in-patient database of the First Affiliated Hospital of Zhejiang Chinese Medicine University from June 2012 to June 2014, 37 eligible patients with Association Research Circulation Osseous stage I or II ONFH who were receiving intra-arterial infusion of auto-PBSCs were recruited. A case form was designed to retrieve relevant data. Hip-preservation failure was defined as the endpoint. All participants were stratified by the categorical risk of collapse, which was statistically tested through log-rank analysis. All significant factors were evaluated using Cox proportional hazards regression model, and a predictive nomogram plot was generated.

**Results:**

In total, 47 hips were followed up for 53.96 ± 21.09 months; the median survival time was 60.18 months. Among the predictors, body mass index (BMI; *P* = 0.0015) and Harris hip score (HHS; *P* < 0.0001) independently affected femoral head survival. Patients with BMI ≥ 24 kg/m^2^ exhibited a 2.58 times higher risk of hip-preservation failure [95% confidence interval (CI), 1.32–5.45] than those with BMI < 24 kg/m^2^, whereas those with HHS ≥ 70 exhibited a 0.19 times lower risk (95% CI, 0.09–0.38) than those with HHS < 70. Hazard ratios associated with age (*P* = 0.042), BMI (*P* = 0.012), HHS (*P* = 0.022), and necrotic volume (*P* = 0.000) were 1.038 (95% CI, 1.001–1.075), 1.379 (95% CI, 1.072–1.773), 0.961 (95% CI, 0.928–0.994), and 1.258 (95% CI, 1.120–1.412), respectively. A nomogram plot (score test *P* = 0.000; C-index = 0.8863) was available for the orthopedic doctor to predict hip survival probability.

**Conclusions:**

The results suggest that intra-arterial infusion of auto-PBSCs prolongs femoral head survival. Age, BMI, HHS, and necrotic volume can influence the efficacy of this intervention.

This study was approved by ethics committee of the trial center, number 2019-KL-075-01.

## Background

Osteonecrosis of the femoral head (ONFH) is a debilitating disease that progresses due to an inadequate blood supply and elevated intraosseous pressure, subsequently leading to bone marrow and osteocyte death [1]. The disease primarily affects patients aged 30–50 years, with a considerable annual incidence [2]; furthermore, it is irreversible. Once the collapse of the femoral head occurs, secondary degeneration of the hip ensues. Eventually, total hip arthroplasty (THA) becomes the inevitable treatment option for the patients [3]. Due to the complications of THA and the limitation of prosthesis durability, optimizing efficient hip-preservation therapy in the early stage is crucial [4].

Although not yet universally accepted, percutaneous bone marrow-derived mesenchymal stromal stem cell (MSC) injection has emerged as an efficient strategy and has a widespread use before collapse; it simultaneously promotes osteogenesis and angiogenesis [5]. A review of the current literature shows that autologous peripheral blood stem cells (auto-PBSCs) likewise contain CD34+ cells (CD34 is an antigen expressed on stem and progenitor cells) [6], which have been proved to a feasible therapy as MSCs [7]. Moreover, auto-PBSCs are easy to collect and safe to use with less complications and lead to an early patient discharge. However, the use of auto-PBSCs remains controversial with regard to the cost of auto-PBSC protocol and characterization of stem cells [8].

This study therefore asked the following questions: (1) Does auto-PBSC transplantation retard the progression of femoral head collapse? (2) What is the risk of hip-preservation failure? (3) How can a predictive model of femoral head survival be achieved after the intervention? (4) What is the specific clinical indication for auto-PBSC transplantation?

## Methods

### Study design and patients

**Fig. 1:**
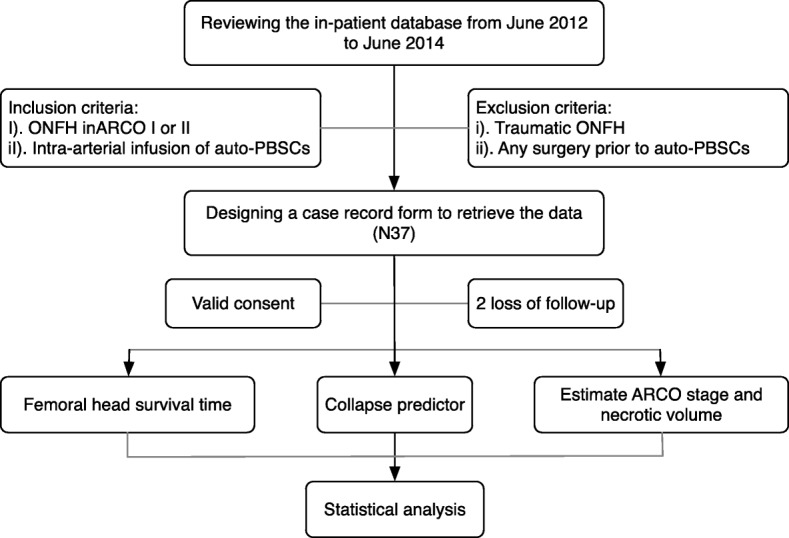
Flowchart of the study

**Fig. 2:**
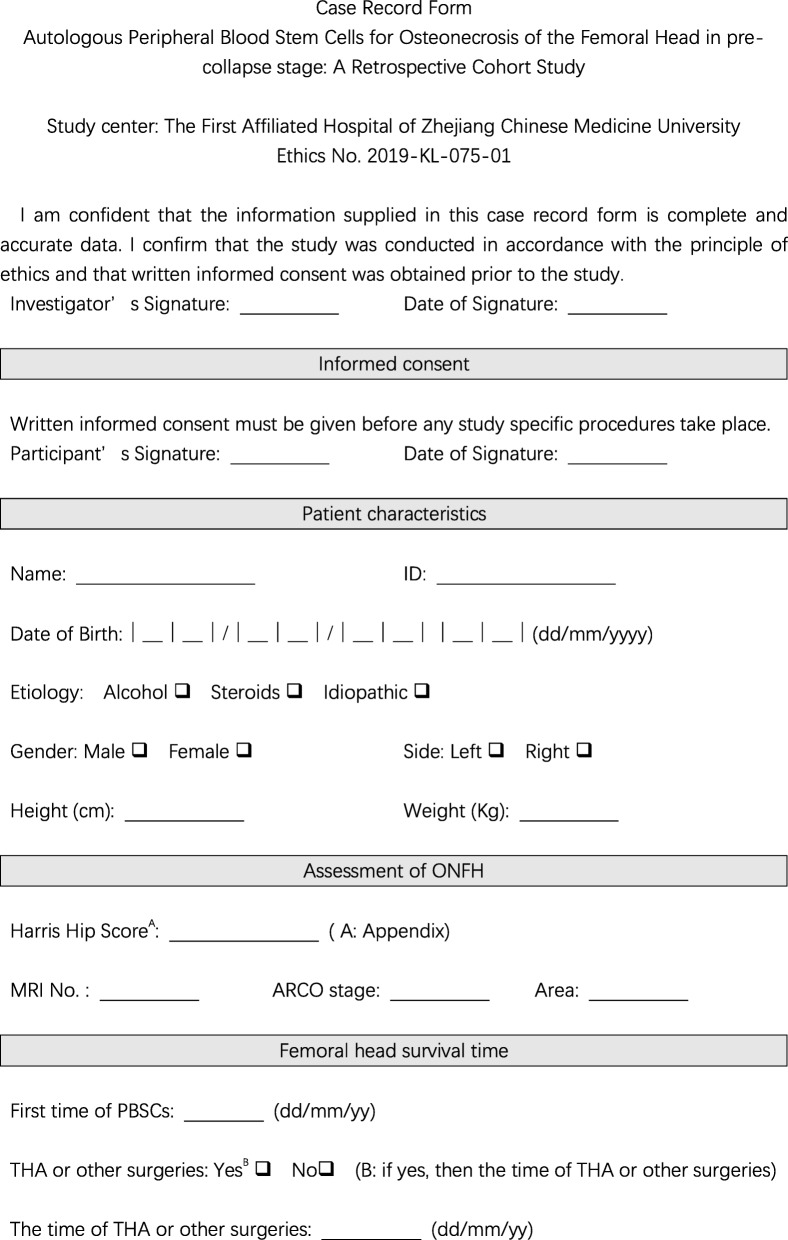
A case record form

This retrospective longitudinal cohort study conforms to the STROBE (strengthening the reporting of observational studies in epidemiology) statement (Fig. 1) and was conducted at the First Affiliated Hospital of Zhejiang Chinese Medicine University. After reviewing the in-patient database from June 2012 to June 2014, 37 patients (2 lost to follow-up) were screened according to the inclusion and exclusion criteria. Briefly, ruling out post-traumatic patients, patients with Association Research Circulation Osseous (ARCO) stage I and II ONFH, those without any history of surgery but intra-arterial infusion of auto-PBSCs, and those with no contraindication for stem cell transplantation were enrolled. Mobilization, collection, and infusion of auto-PBSCs were performed three times every month. One researcher was responsible for data collection that was based on a case record form (Fig. 2); this form was primarily designed to obtain written informed consent and to retrieve data, including patient characteristics, hip function, and femoral head survival time. Furthermore, one researcher was assigned to read magnetic resonance imaging (MRI) findings and measure necrotic volume, and another one performed statistical analysis. This study was approved by ethics committee of the trial center, number 2019-KL-075-01.

### PBSC mobilization, collection, enumeration, and transplantation

**Fig. 3:**
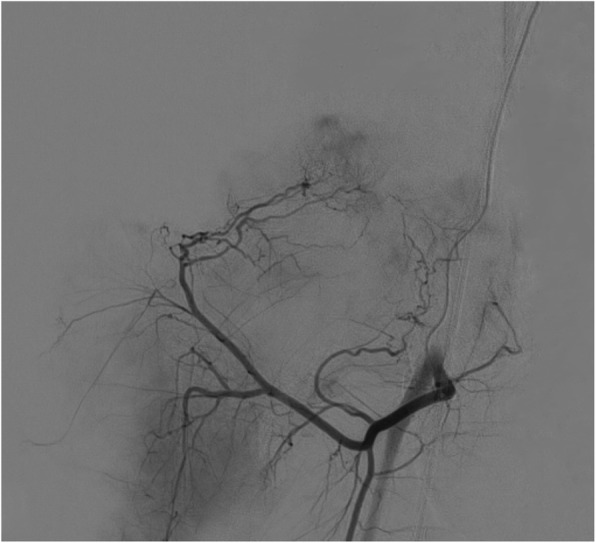
Auto-PBSCs were transplanted into the femoral head in DSA

Patients were intravenously injected with recombinant human granulocyte colony stimulating factor (5 μg/kg/day) for 5 days. On day 6, auto-PBSCs were harvested using COBE Spectra (COBE BCT, Inc., Lakewood, CO, USA) individually based on weight, height, red blood cell volume ratio, and PBSC volume. A formula, i.e., VPBSCs × countingWBC × PCTMO/weight (*V*_PBSCs_ for the volume of PBSCs, PCT_MO_ for percent of mononuclear), was used to assess the activity of PBSCs; the desired result was a value above (5–8) × 10^8^/kg [9]. Once enumeration was approved, auto-PBSCs with iodinate contrast agent were infused through the arteria profundal femoris to the arteria circumflex femoris medialis and lateralis using Seldinger technique in digital subtraction angiography (DSA) as shown in Fig. 3.

### Data assessment

A total of 35 patients (47 hips) from whom a valid consent was obtained were followed up. Data were collected based on the translated case record form, mainly outlining demographic information (gender, age, height, and weight), ONFH condition [etiology, unilateral/bilateral, and Harris hip score (HHS)], imagistic information (MRI number), and femoral head survival time (in months); the endpoint was hip-preservation failure, defined as conversion to total hip arthroplasty (THA) or other surgeries (core decompression ± non-vascularized/vascularized bone graft ± biological agents or osteotomy). Simultaneously, MRI (GE Medical Systems, Milwaukee, WI, USA) was performed to differentiate ARCO stages (Ia, Ib, Ic, IIa, IIb, or IIc), and the necrotic area of each coronal and transverse T2 slice was blindly and repeatedly measured three times using Fiji ImageJ 2.0. Subsequently, necrotic volume was calculated by multiplying the thickness of each slice.

### Statistical analysis

Data analysis was performed using SPSS 25.0 (IBM, Armonk, NY, USA), GraphPad Prism 6.0 (GraphPad, San Diego, CA, USA), and R language. Baseline characteristics of the patients were represented by descriptive statistics comprising mean ± standard deviation (SD) for continuous variables and frequency count (percentage) for categorical data. The Kaplan-Meier method was used to plot the overall femoral head survival curve with median survival time and number at risk table. Log-rank analysis was used to determine the differences in variates, including gender, age (≤ 40 vs. > 40 years), body mass index (BMI; < 24 vs. ≥ 24 kg/m^2^), HHS (< 70 vs. ≥ 70), and ARCO stage (I vs II); significance level was set at *P* < 0.15 for those. With variance inflation factor (VIF) of independent variables estimating collinearity and score test confirming the model effect, all predictors (*P* < 0.05) with adjusted hazard ratio (HR) and 95% confidence interval (CI) were assessed using the Cox proportional hazards regression model with the enter method. Concordance index (C-index; range, 0.5–1.0) was calculated to determine the discriminatory property of the Cox model. Finally, a nomogram plot was drawn to predict 1-, 3-, 5-, and 7-year hip-preservation probabilities.

## Results

### Study population

Between June 2012 and June 2014, 37 patients were screened to be eligible, but 1 patient disconnected and 1 refused to participate (2 lost to follow-up). Thus, 35 participants (47 hips) were followed from the time of initial intervention until the endpoint of hip-preservation failure or until July 2019 when the study was performed. Patient characteristics are summarized in Table 1. The cohort, with 47 hips as the subjects, comprised 10 censored data (1 patient has died and 9 subjects did not meet the endpoint). Eventually, the entire follow-up time ranged from 8 to 84^+^ months (53.96 ± 21.09 months). Life table demonstrated that the 1-, 3-, 5-, and 7- year cumulative probabilities of survival were 96 ± 3%, 81 ± 6%, 50 ± 7%, and 12 ± 6%, respectively, with a median survival time of 60.18 months.

**Table 1:** Baseline characteristics of enrolled patients

	*N* = 35
Female to male	15:20
Age (years)	39.06 ± 12.53
BMI (kg/m^2^)	24.27 ± 2.07
Weight (kg)	68 ± 8.35
Height (m^2^)	1.67 ± 0.08
Etiology (%)	
Alcohol	6 (17.14)
Steroids	6 (17.14)
Idiopathic	23 (65.71)
Unilateral to bilateral	13:12 (*n* = 47^a^)
Harris hip score	61.97 ± 14.71
ARCO stage	
Ia:Ib:Ic	5:08:06
IIa:IIb:IIc	9:12:07
Volume of lesion (cm^3^)	13.63 ± 6.11

^a^The number of hips with ONFH

### Candidate predictors

**Fig. 4:**
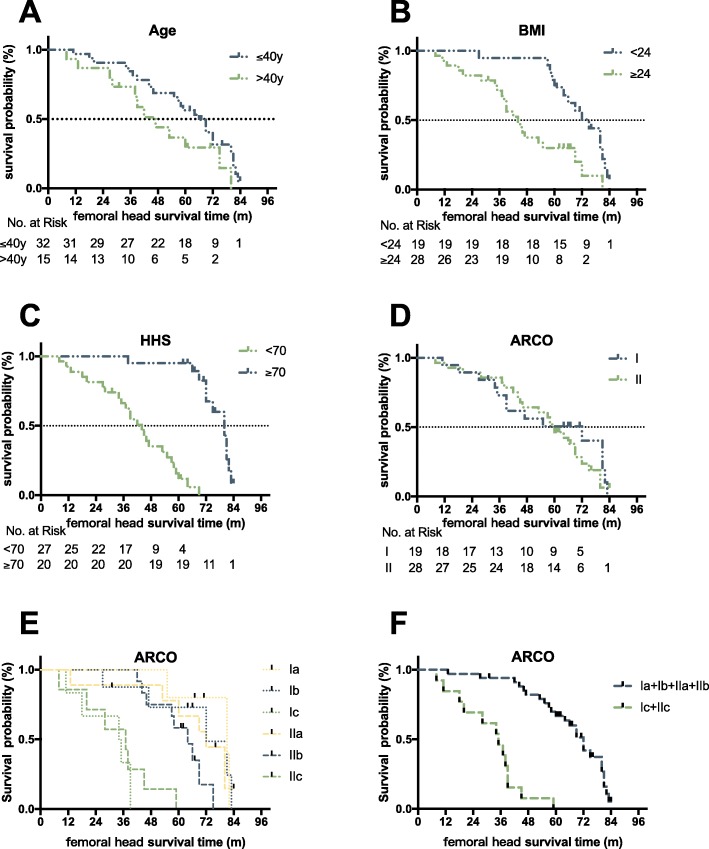
Kaplan-Meier survival curves showing femoral head survival dependent on age (**a**), BMI (**b**), HHS (**c**), and ARCO (**d**, **e**, **f**)

Regarding the relative risk factors, gender, age, BMI, HHS, ARCO stage, and necrotic volume were all statistically analyzed through log-rank analysis (*P* < 0.15; Fig. 4). Age (*P* = 0.073), BMI (*P* = 0.0015), HHS (*P* < 0.0001), and necrotic volume were identified as preliminary predictors of femoral head collapse after auto-PBSC transplantation, whereas gender (*P* = 0.26) and ARCO stage (*P* = 0.49) were not. However, even if ARCO I versus II was additionally precluded from the log-rank analysis due to the violation of proportional hazards assumption, ARCO stages Ia, Ib, IIa, and IIb were significantly different from ARCO stages Ic and IIc (*P* < 0.0001), which are sub-grouped by the extent of involvement; this ensured that necrotic volume was correlated to femoral head survival. Compared with patients with BMI < 24 kg/m^2^, those with BMI ≥ 24 kg/m^2^ had a significant risk of hip-preservation failure (HR, 2.58; 95% CI, 1.32–5.45; *P* = 0.0015), whereas HHS ≥ 70 was a notable protective factor (HR, 0.19; 95% CI, 0.09–0.3; *P* < 0.0001; Table 2). Incidentally, either BMI or HHS was yet a univariate effector, so Cox regression models were analyzed to determine association with age and necrotic volume.

**Table 2:**
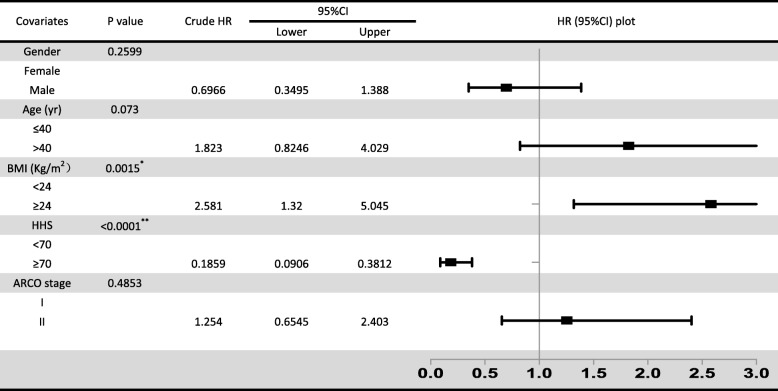
Univariate log-rank analysis of categorical risk factors

*BMI and **HHS (*P* < 0.05) were independent predictors

### Cox regression model

**Fig. 5:**
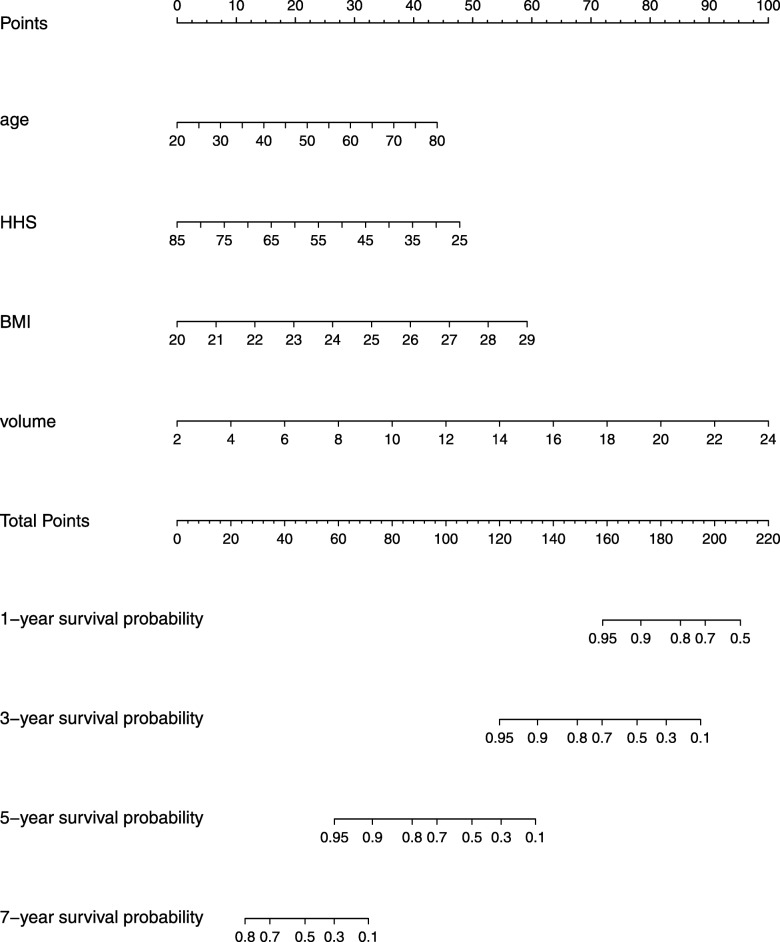
Nomogram plot of 1-, 3-, 5-, and 7-year survival probability according to age, BMI, HHS, and femoral head necrotic volume

Based on linear regression, age, BMI, HHS, and necrotic volume presented no collinearity (VIF < 3). The multivariate effect on time to femoral head collapse (Table 3) showed that age (*P* = 0.042), BMI (*P* = 0.012), HHS (*P* = 0.022), and necrotic volume (*P* = 0.000) were significant predictors. Especially regarding necrotic volume, it was suggested that, for every 1-cm^3^ increase in the volume, the hazard increased by 25.8% (95% CI, 12–41.2). The effect of the model was significant (score test *P* = 0.000). Furthermore, a discriminating nomogram plot (Fig. 5) was generated to predict 1-, 3-, 5-, and 7-year hip-preservation probabilities (C-index = 0.8863). For the orthopedic doctor, the plot was available to locate a patient’s age, BMI, HHS, and necrotic volume in each axis; to draw a line straight upward to the point axis and sum up the total points; and then to draw a line straight downward to determine the patient’s 1-, 3-, 5-, and 7-year femur head survival after auto-PBSC transplantation.

**Table 3:**
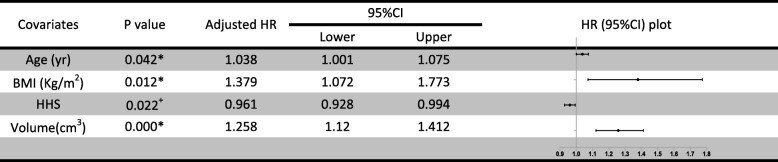
Multivariate Cox regression analysis of continuous risk factors

*Age, BMI, and volume were significant risk, while ^+^HHS was a protector

### Complications

There was no complication that occurred during auto-PBSC mobilization, collection, and infusion.

## Discussion

ONFH is a progressive disease that inevitably causes femoral head collapse and hip osteoarthritis. Consequently, it is crucial to retard the progression; however, there is no consensus regarding the treatment of ONFH in the pre-collapse stage. Recent literature suggests stem cell transplantation, with the potential of osteogenesis and angiogenesis, as a feasible and safe intervention for ONFH [10, 11]. MSCs comprising osteogenic progenitor cells were initially applied for ONFH in 1989 [12] and were proven to efficiently delay THA. Alternatively, PBSCs show superiority over MSCs [13] because PBSCs are easier to collect and their transplantation is associated with less complications and an earlier patient discharge. In contrast, MSCs commonly derived from the bone marrow, where tumor cells may reside, requiring culturing ex vivo that incurs extra cost. However, numerous controversies remain regarding the use of PBSCs, which gives rise to the question of what are the exact indications for the use of PBSCs. To the best of the author’s knowledge, this has not been studied until now; hence, this study was designed to achieve a predictive model after auto-PBSC transplantation, more specifically to identify the indications for this intervention.

In this study, no complication occurred during auto-PBSC mobilization, collection, and infusion, proving that it is a safe intervention for patients with ONFH. As PBSCs, theoretically, have the potential to differentiate into vascular endothelial cells and secrete biologically active paracrine factors for regeneration [14, 15], this study cohort, exhibiting a 50 ± 7% hip-preservation success rate at 5-year follow-up, supports the efficacy of auto-PBSC transplantation for ONFH compared with previous studies, which suggested that > 50% of patients with ONFH required THA within 3 years of diagnosis [16, 17]. However, the outcomes were individually different; therefore, the factors that led to this difference were investigated. First, etiology was revealed as a predictor [18, 19], but in the present study, it was unlikely significant and violated the proportional hazards assumption. Gender (*P* = 0.2599), age (*P* = 0.0730), BMI (*P* = 0.0015), HHS (*P* < 0.0001), and ARCO stage (*P* = 0.4853) were than analyzed as independent risk factors for progression. As a result, BMI and HHS were identified as potent factors, significantly supporting the efficacy of auto-PBSCs. Finally, as age (*P* < 0.15) was related to auto-PBSC differentiation capacity [9, 20] and as the size of lesion was recognized as a specific predictor that has an enormous impact on femoral head collapse [21], a predictive model containing age (*P* = 0.042), BMI (*P* = 0.012), HHS (*P* = 0.022), and necrotic volume (*P* = 0.000) was established. The model exhibited excellent discrimination (C-index = 0.8863) and calibration (score test *P* = 0.000). Absolute necrotic volume (*P* = 0.000) was a notable risk factor (HR, 1.258; 95% CI, 1.120–1.412). Both age (HR, 1.038; 95% CI, 1.001–1.075) and BMI (HR, 1.379; 95% CI, 1.072–1.773) were also predictors of time to hip-preservation failure after the intervention. Furthermore, BMI, compared with age and necrotic volume, is variable; thus, it is reasonable to advise patients, particularly those who are overweight, to be fit. In contrast, HHS was identified as a protector (HR, 0.961, 95% CI, 0.928–0.994), indicating that fair HHS was associated with a favorable outcome.

Simultaneously, this study has certain limitations and strengths. It was a retrospective single-center study that has an inherent risk of bias (recall bias) and confounders (no wash-out period), resulting in an insufficient level of evidence compared with a randomized controlled trial. Concurrently, the author’s original research was reproducible to identify the potential survival probability in 1–7 years, which has not been given forth, and will mainly assist the clinical decision on auto-PBSCs. In the future, the prediction model should be prospectively substantiated. Moreover, it is doubtful if the efficacy of other interventions would be proven with every risk under control.

## Conclusions

Auto-PBSC transplantation is a considerable promising treatment to prolong hip preservation. Age, BMI, HHS, and necrotic volume are the predictors of 5-year femoral head survival probability after auto-PBSC transplantation.

## Supplementary information

**Additional file 1. ***No.* represents the number of hip. To gender, *1* represents male and *0* stands for female. Age means how old the patient is when the intervention takes place. BMI is patient’s body mass index. HHS is Harris hip score. ARCO is the stage of ONFH based on the MRI and criteria of ARCO, consisting *1* for Ia, *2* for Ib, *3* for Ic, *4* for IIa, *5* for IIb and *6* for IIc. Time is the length of survival time of femoral head, recorded by month. Status means whether meets the endpoint, *0* for censor and *1* for hip-preserving failure.

## Data Availability

The dataset supporting the conclusions of this article is included within the article.

## Abbreviations

ARCO: Association Research Circulation Osseous
Auto-PBSC: Autologous peripheral blood stem cell
BMI: Body mass index
CI: Confidence interval
C-index: Concordance index
DSA: Digital subtraction angiography
HHS: Harris hip score
HR: Hazard ratio
MRI: Magnetic resonance imaging
MSC: Mesenchymal stromal stem cell
ONFH: Osteonecrosis of the femoral head
SD: Standard deviation
STROBE: Strengthening the reporting of observational studies in epidemiology
THA: Total hip arthroplasty
VIF: Variance inflation factor
